# Book Reviews

**Published:** 1898-12

**Authors:** 


A Manual of General Pathology. For Students and Practitioners. By Walter Sidney Lazarus-Barlow, M. D., M. R. C. P., late Demonstrator of Pathology in the University of Cambridge, etc. Octavo, pp. xi.—795. Philadelphia: P. Blakiston's Son & Co. 1898.

This work must be estimated for its handling of the subject of general pathology, and not confounded with the usual treatise on pathological anatomy. The latter receives much completer attention at the hands of teachers than the former, hence, as a rule, students acquire more knowledge of morbid anatomy than they do of general pathology. The author is most conscientious in handling his subject, and gives all the most recent references at the close of his chapters.

This, then, is essentially a treatise on the pathology of function rather than that of structure, hence deserves the most careful study by the student who would acquire the best knowledge. The author's chapter on vegetable microörganisms is well written, and that on the pathology of inflammation has been only rarely equaled. His style is attractive, and his rhetoric charming. These are important, for a dry subject clothed in an elegant diction becomes at once a pleasant study, whereas otherwise it might be neglected.

The general pathology of new growths as set forth in this book deserves the highest praise. Moreover, it is one of the important fields for a student to pursue, for some phase of the subject is presented in every-day practice. That part of the book devoted to the consideration of morbid secretions and excretions, too, is deserving of careful study on account of its practical bearing on patients met almost daily by the general physician.

Taken all in all we have not lately met a book that has so nearly filled "a long felt want" as does this one, and we have nothing but praise to offer in its behalf. We should like to see it in the library of every practising physician.

A Text-book upon the Pathogenic Bacteria. For Students of Medicine and Physicians. By Joseph McFarland, M. D., Professor of Pathology in the Medico-Chirurgical College, Philadelphia, etc., etc. Octavo, pp. 497. With 134 illustrations. Second edition, revised and enlarged. Philadelphia: W. B. Saunders. 1898.

This work, even in its self-limited scope, is one of the most satisfactory bacteriological text-books with which we are familiar. It does not cover the whole field of parasitology, but it does describe in scientific detail such bacteria as lesions or toxins have proven to be of a pathogenic character. This was the original design of the author and he has not materially changed his plan in this edition, though he has added considerably to the amount of technique which the former contained, hence it is made to fill the dual rôle of a systematic work upon bacteria and a laboratory guide as well. Some new chapters have also been added relating to the bacteriology of mumps, whooping cough, yellow fever, and the like; also describing methods of determining the value of antiseptics and germicides and of determining the thermal death-point. The bacillus of tuberculosis receives, as might be expected, ample study, and the author proves most interesting in its consideration. The illustrations are, for the most part, of good quality, but their number might have been increased with benefit. Students will find this an excellent guide to bacteriological research and practitioners will gain in its pages just the information they need to aid in diagnosis.

---

Seventeenth Annual Report of the State Board of Health of New York. Transmitted to the Legislature February 15, 1897. New York and Albany: Winkoop, Hallenbeck, Crawford Co., State Printers. 1898.

From this report we learn that there were 124,600 deaths in the state during the period which it covers, viz., the year 1896. This is a death-rate of 18.50 per thousand population. Of the deaths 28.5 per cent. occurred in the summer months, 25.7 in the spring, 24.3 in the winter, and 21.5 in the autumn. This is contrary to the popular belief, which accords the heaviest death-rate to the winter season. According to the report there has been during the last few years a steady diminution in the number of deaths from consumption in the maritime district, which includes the territory of Greater New York. This is solely attributable to the fact that the local boards of health in that district have recognised that the disease is infectious, and have taken rigid precautions to prevent its spread.

The present State Board of Health, under the presidency of Dr. Daniel Lewis, is doing efficient service, and should be supported in its endeavors to prevent the spread of disease and to limit and reduce the death-rate by all the power the executive, legislative and judicial branches of the state government can wield.

Twenty-Second Year Book of the New York State Reformatory. For the fiscal year ending September 30, 1897. With illustrations and tables. Elmira. N. Y. 1898.

This interesting volume consisting of 148 octavo pages, amply illustrated and well bound is, in editing, typography, illustrations and binding, solely the product of the labor of the inmates of the institution. The report shows that of an average number of inmates, 1,498, there were admitted to the hospital during the year 232. Of this number sixteen died, ten deaths being due to consumption.

The reformatory has reached its limit of population and additional accommodations are urgently demanded. The legislature should not be slow or illiberal in dealing with the requirements of this excellent institution.

TWENTY-FOURTH ANNUAL REPORT of the Secretary of the State Board of Health of the State of Michigan for the Fiscal Year ending June 30, 1898. Lansing. 1897.

Seven years before New York had a state board of health Michigan could boast of one of the best organised boards in this country. Dr. Henry B. Baker, of Lansing, is its secretary and it is doubtful if any state has in service a better sanitary officer. These reports are among the best issued, and in addition weekly, monthly and quarterly bulletins are sent out by the secretary, hence the people are kept advised of everything of importance pertaining to sanitary affairs within the borders of the state. The volume under consideration is full of interesting material for sanitarians and public health officers.

---

ATLAS AND EPITOME OF OPERATIVE SURGERY. By DR. OTTO ZUCKERKANDI, Privatdocent in the University of Vienna. Authorised translation from the German. Edited by J. CHALMERS DACOSTA, M. D., Clinical Professor of Surgery in Jefferson Medical College, Philadelphia, etc. Duodecimo, pp. 395. With twenty-four colored plates and 217 illustrations in the text. Philadelphia: W. B. Saunders. 1898.

This atlas is one of an excellent series and in and of itself possesses much merit. It is, as the author himself states, intended as an elementary work for students in the subject, but we see a useful place for it in the library of a physician who does not practise surgery daily and needs, on occasions, a ready reference volume of anatomical landmarks and surgical technique. The colored plates are especially clear, and the text is concise as well as lucid. We regard it as an exceedingly useful book.

---

THE MEDICAL NEWS VISITING LIST FOR 1899. Weekly (dated for 30 patients); Monthly (undated, for 120 patients per month); Perpetual (undated for 30 patients weekly per year); and Perpetual (undated for 600 patients weekly per year). The first three styles contain 32 pages of data and 160 pages of blanks. The 60-patient Perpetual consists of 256 pages of blanks. Each style is in one wallet-shaped book, with pocket, pencil and rubber. Seal grain leather, $1.25. Thumb-letter index, 25 cents extra. Philadelphia and New York: Lea Brothers & Co.

This visiting list has been a favorite with the profession for some years, combining as it does so many good qualities. It is practical, convenient in size, well made and tastefully bound. In these days no physician can dispense with the use of a visiting list, hence it is only a question of which one he shall purchase. In addition to the usual daily record of patients this one contains among other things thirty-two pages of printed data of the most useful sort, including an alphabetical table of diseases with approved remedies, a table of doses, sections on examination of urine, artificial respiration, incompatibles, poisons and antidotes, a diagnostic table of eruptive fevers, and a full-page plate, showing at a glance the incisions for ligation of the various arteries, a valuable guide in such emergencies. Those physicians who have heretofore provided themselves with this book will be likely to continue to do so and others who may purchase it for the first time will not be disappointed in its usefulness.

---

THE PHYSICIAN'S VISITING LIST (LINDSAY & BLAKISTON'S) FOR 1899. Forty-eighth year of its publication. Sold by all booksellers and druggists. Philadelphia: P. Blakiston's Son & Co. 1898.

This visiting list is an old friend that appears with regularity and always in good form. Its table of contents embraces, inter alia, the metric system of weights and measures, table for comparative apothecary's weights and measures into grains, dose table, instructions for treatment of asphyxia and apnea, comparison of thermometers, and a table for calculating the period of utero-gestation. Besides these there is a variety of useful material and information desirable to have for handy reference. It is beautifully printed and durably bound. It is published in three forms—regular edition for from 25 to 100 patients a week, price, $1.00 to $2.25—perpetual edition, same as regular without dates for 1,300 to 2,600 names, $1.25 to $1.50—and monthly edition, which can be commenced at any time, price, 75c. to $1.00. We commend this excellent book to the busy practitioners of medicine as one of the most useful of its kind.

